# Peripheral blood AKAP7 expression as an early marker for lymphocyte-mediated post-stroke blood brain barrier disruption

**DOI:** 10.1038/s41598-017-01178-5

**Published:** 2017-04-26

**Authors:** Grant C. O’Connell, Madison B. Treadway, Ashley B. Petrone, Connie S. Tennant, Noelle Lucke-Wold, Paul D. Chantler, Taura L. Barr

**Affiliations:** 1 Center for Basic and Translational Stroke Research, Robert C. Byrd Health Sciences Center, West Virginia University, Morgantown, West Virginia USA; 2 Department of Pharmaceutical Sciences, School of Pharmacy, West Virginia University, Morgantown, West Virginia USA; 3 Department of Biology, Eberly College of Arts and Sciences, West Virginia University, Morgantown, West Virginia USA; 4 Center for Cardiovascular and Respiratory Sciences, Robert C. Byrd Health Sciences Center, West Virginia University, Morgantown, West Virginia USA; 5 Division of Exercise Physiology, School of Medicine, West Virginia University, Morgantown, West Virginia USA; 6 Valtari Bio Incorporated, Morgantown, West Virginia USA

## Abstract

Our group recently identified 16 genes whose peripheral blood expression levels are differentially regulated in acute ischemic stroke. The purpose of this study was to determine whether the early expression levels of any of these 16 genes are predictive for post-stroke blood brain barrier (BBB) disruption. Transcriptional expression levels of candidate genes were measured in peripheral blood sampled from ischemic stroke patients at emergency department admission, and BBB permeability was assessed at 24 hour follow up via perfusion-weighted imaging. Early heightened expression levels of *AKAP7*, a gene encoding a protein kinase A-binding scaffolding molecule, were significantly associated with BBB disruption 24 hours post-hospital admission. We then determined that *AKAP7* is predominantly expressed by lymphocytes in peripheral blood, and strongly co-expressed with *ITGA3*, a gene encoding the adhesion molecule integrin alpha 3. Subsequent *in vitro* experiments revealed that heightened expression of *AKAP7* and *ITGA3* in primary human lymphocytes is associated with a highly adherent phenotype. Collectively, our results suggest that *AKAP7* expression levels may have clinical utility as a prognostic biomarker for post-stroke BBB complications, and are likely elevated early in patients who later develop post-stroke BBB disruption due to the presence of an invasive lymphocyte population in the peripheral blood.

## Introduction

The inflammatory state which persists at the level of the neurovascular unit following ischemic stroke can drive delayed disruption of the blood brain barrier (BBB) within and around the infarcted region. Heightened activity of proteinases disrupt the pericellular junctions of the endothelium and allow for migration of peripheral immune cells into the damaged brain parenchyma; this process inherently compromises the integrity of the BBB and increases the risk of edema and hemorrhagic transformation^1, 2^. By nature, administration of thrombolytic therapies can exacerbate the risk of such adverse events and worsen outcome in patients who develop complications^3–5^. Early identification of patients who are at risk for the development of BBB complications can inform acute clinical decisions regarding thrombolytic intervention, and ultimately improve clinical management and patient outcome^6^. Unfortunately, the diagnostic tools presently available to clinicians to identify patients at risk for post-stroke BBB complications are limited.

Currently, perfusion-weighted magnetic resonance imaging (MRI) using gadolinium-diethylene triamine penta-acetic acid (Gd-DTPA) contrast is one of the most sensitive methods to detect early changes in BBB permeability in the clinical setting. The intact BBB is largely impermeable to Gd-DTPA, thus, hyperintense post-contrast enhancement of the cerebrospinal fluid (CSF) space on fluid-attenuated inversion recovery (FLAIR) is indicative of BBB disruption^7^. Such post-contrast enhancement has been labeled as hyperintense acute reperfusion injury marker (HARM)^8^, and has been shown to be predictive of post-stroke edema formation and hemorrhagic transformation^8, 9^. While perfusion-weighted imaging can yield valuable diagnostic information which can be used to guide clinical care decisions^6, 10, 11^, a large number of healthcare facilities lack the capacity to perform MRI during acute triage^12^. As a result, there has been a push for the identification of peripheral blood biomarkers which can provide similar insight regarding the status of the BBB during the acute phase of care.

It is well known that the transcriptome of the peripheral immune system responds rapidly to ischemic brain injury^13–16^. Because the peripheral immune system plays a critical role in the pathogenesis of BBB disruption^2, 17^, it is possible that there are early transcriptional changes in the peripheral immune system which could be used to identify patients at high risk of developing post-stroke BBB complications^18^. Ideally, to be clinically useful, such transcriptional biomarkers would be specific to stroke pathology. Our group recently identified a panel of 16 genes (*ACSL1*, *AKAP7*, *APOBEC3A*, *ARG1*, *CA4*, *CCR7*, *CRISPLD2*, *CSPG2*, *FCGR3B*, *FOLR3*, *IQGAP1*, *LY96*, *MMP9*, *ORM1*, *PADI4*, *S100A12*) which exhibit stroke-specific differential regulation in peripheral whole blood^14^. The purpose of this study was determine whether the early expression levels of any of these 16 genes are predictive for post-stroke BBB disruption, and if so, to determine their role in the pathogenesis of BBB breakdown.

## Results

### Early peripheral blood expression of AKAP7 is associated with the development of post-stroke BBB disruption

**Figure 1:**
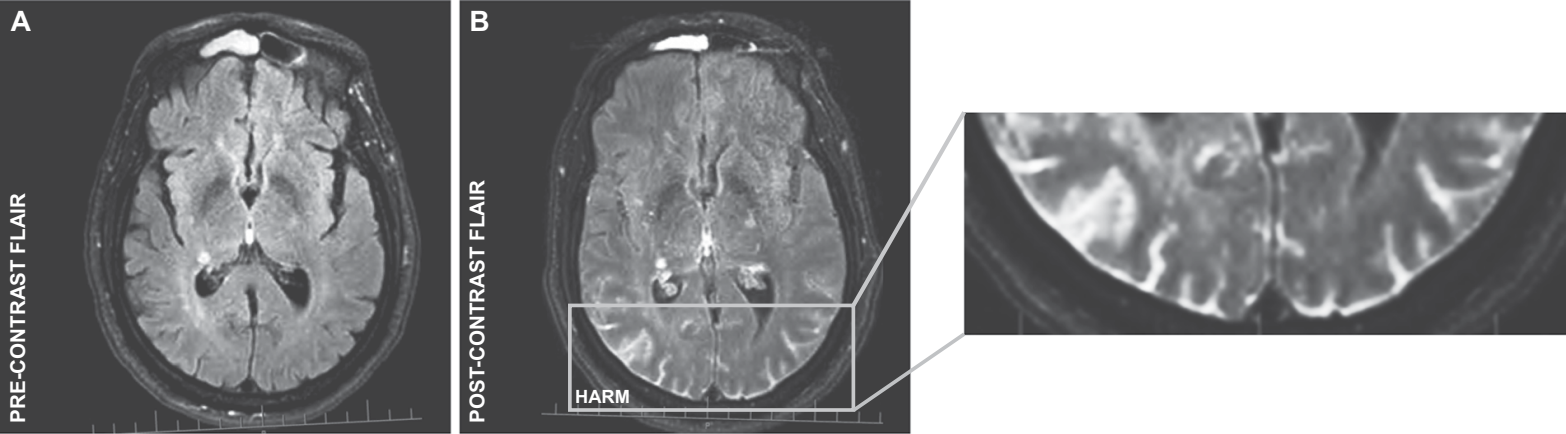
Assessment of post-stroke BBB disruption via HARM on perfusion-weighted imaging. (**A**) Pre-contrast and (**B**) post-contrast FLAIR images from a patient presenting with HARM at 24 hour follow up. HARM was positively identified when the CSF space in the sulci or ventricles appeared hyperintense post-contrast. Level of HARM was systematically categorized as none, mild, moderate, or severe based on the number of serial slices exhibiting evidence of HARM.

In order to determine the relationships between the expression levels of the 16 stroke-associated candidate genes and post-stroke BBB disruption, we first recruited a modestly sized discovery cohort consisting of 27 acute ischemic stroke (AIS) patients. Peripheral whole blood samples were obtained at emergency department admission and transcriptional profiling was performed via microarray. BBB permeability was assessed via level of HARM (none, mild, moderate, or severe) on perfusion-weighted imaging at 24 hour follow up (Fig. 1). We then employed bias-reduced logistic regression using forward stepwise variable selection to determine whether early expression levels of any the 16 candidate genes were predictive for the development of post-stroke severe HARM.

The median time from symptom onset to blood draw across all discovery cohort subjects was 7.4 hours. In terms of clinical and demographics characteristics, patients presenting with severe HARM at 24 follow up were significantly older and displayed a higher prevalence of dyslipidemia relative to patients presenting with lower levels of HARM (Table 1). Furthermore, a higher proportion of patients presenting with severe HARM received thrombolytic intervention via recombinant tissue plasminogen activator (rtPA) than those presenting with lower levels of HARM, however this difference was not statistically significant.

**Table 1:** Discovery cohort clinical and demographic characteristics.

	HARM CATEGORY:				STAT (df)	p
	NONE (n = 4)	MILD (n = 8)	MODERATE (n = 7)	SEVERE (n = 8)		
^a^Age (*mean* ± *SD*)	53.5 ± 11.5	74.1 ± 9.6	74.4 ± 11.3	75.9 ± 15.4	f = 3.45 (3, 23)	0.033*
^b^Female *n* (%)	3 (75.0)	4 (50.0)	3 (42.9)	4 (50.0)	χ^2^ = 1.11 (3)	0.775
^a^Baseline NIHSS (*mean* ± *SD*)	2.5 ± 1.9	1.9 ± 2.9	6.1 ± 5.7	5.8 ± 6.2	f = 1.89 (3, 23)	0.159
^b^Partial anterior cerebral infarction *n* (%)	2 (50.0)	3 (37.5)	4 (57.1)	5 (62.5)	χ^2^ = 2.61 (3)	0.456
^b^Total anterior cerebral infarction *n* (%)	0 (00.0)	0 (0.00)	1 (14.3)	0 (00.0)	χ^2^ = 1.75 (3)	0.627
^b^Lacunar cerebral infarction *n* (%)	1 (25.0)	4 (50.0)	1 (14.3)	3 (37.5)	χ^2^ = 2.33 (3)	0.507
^b^Posterior cerebral infarction *n* (%)	1 (25.0)	1 (12.5)	1 (14.3)	0 (00.0)	χ^2^ = 1.87 (3)	0.601
^b^Hypertension *n* (%)	2 (50.0)	4 (50.0)	5 (71.4)	5 (62.5)	χ^2^ = 0.89 (3)	0.828
^b^Diabetes *n* (%)	0 (00.0)	2 (25.0)	0 (00.0)	2 (25.0)	χ^2^ = 3.23 (3)	0.358
^b^Atrial fibrillation *n* (%)	0 (00.0)	1 (12.5)	0 (00.0)	1 (12.5)	χ^2^ = 1.46 (3)	0.686
^b^Myocardial infarction *n* (%)	0 (00.0)	2 (25.0)	0 (00.0)	1 (12.5)	χ^2^ = 2.95 (3)	0.399
^b^Dyslipidemia *n* (%)	0 (00.0)	5 (62.5)	3 (42.9)	7 (87.5)	χ^2^ = 8.92 (3)	0.030*
^b^Hypertension medication *n* (%)	1 (25.0)	4 (50.0)	7 (100)	6 (75.0)	χ^2^ = 5.09 (3)	0.165
^b^Diabetes medication *n* (%)	0 (00.0)	2 (25.0)	0 (00.0)	0 (00.0)	χ^2^ = 5.13 (3)	0.163
^b^Cholesterol medication *n* (%)	0 (00.0)	5 (62.5)	3 (42.9)	5 (62.5)	χ^2^ = 5.11 (3)	0.164
^b^Anticoagulant/antiplatelet *n* (%)	0 (00.0)	5 (62.5)	4 (57.1)	5 (62.5)	χ^2^ = 5.11 (3)	0.164
^b^rtPA *n* (%)	0 (00.0)	1 (12.5)	3 (42.9)	4 (50.0)	χ^2^ = 4.99 (3)	0.173
^b^Current smoker *n* (%)	1 (25.0)	0 (00.0)	1 (14.3)	0 (00.0)	χ^2^ = 3.50 (3)	0.321
^b^Family History of stroke *n* (%)	1 (25.0)	4 (50.0)	1 (14.3)	4 (50.0)	χ^2^ = 2.96 (3)	0.399
^b^Previous stroke *n* (%)	0 (00.0)	0 (00.0)	3 (42.9)	1 (12.5)	χ^2^ = 6.48 (3)	0.090

Inter-group statistical comparisons were made via ^a^one-way ANOVA or ^b^4 × 2 chi-squared test; SD, standard deviation; df, degrees of freedom; *p < 0.05; NIHSS, National Institutes of Health stroke scale; rtPA, recombinant tissue plasminogen activator.

**Figure 2:**
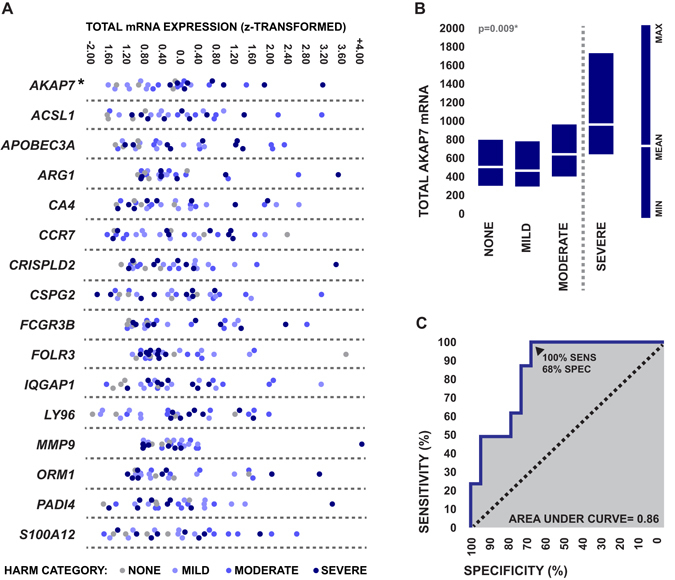
Relationships between the early expression levels of candidate genes and post-stroke BBB disruption. (**A**) Relationships between peripheral blood total transcriptional expression levels of each of the 16 candidate genes at hospital admission and level of HARM on perfusion-weighted imaging at 24 hour follow up. Expression levels are presented as standardized values generated from mean normalized microarray florescence intensities. Significance of relationships between gene expression levels and severe HARM was assessed via bias-reduced logistic regression. (**B**) Relationship between peripheral blood total transcriptional expression levels of AKAP7 at hospital admission and level of HARM on perfusion weighted imaging at 24 hour follow up. Expression levels are presented as mean normalized microarray florescence intensity and were compared between HARM categories using one-way ANOVA. (**C**) Receiver operator curve depicting the sensitivity and specificity of admission peripheral blood total AKAP7 levels as an identifier of patients who later developed severe HARM.

Of the 16 candidate genes, only the total expression levels of *AKAP7*, a gene encoding a protein kinase A (PKA)-binding scaffolding molecule known as A kinase anchoring protein 7 (AKAP7), were significantly associated with the development of post-stroke severe HARM after controlling for age, dyslipidemia, and rtPA (p = 0.025*, Odds ratio = 4.0, 95% confidence interval = 1.1–56.8, Fig. 2A). Specifically, elevated whole blood expression levels of total *AKAP7* at emergency department admission were associated with increased degree of HARM at 24 hour follow up (Fig. 2B). Furthermore, early total expression levels of *AKAP7* showed a modest ability to predict post-stroke BBB disruption, demonstrating 100% sensitivity (95% confidence interval = 63.1–100%) and 68.1% specificity (95% confidence interval = 43.5–87.0%) for the development of severe HARM (Fig. 2C).

### AKAP7 exhibits strong co-expression with ITGA3 in peripheral blood following stroke

**Figure 3:**
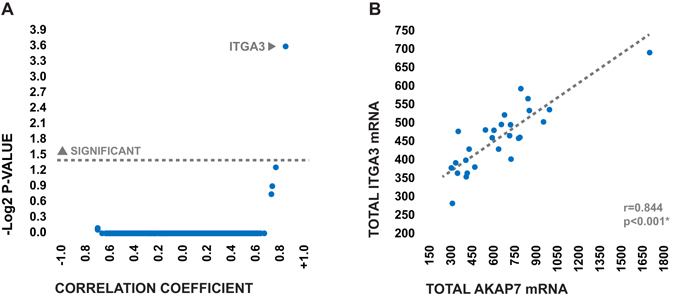
Results of genome-wide correlational analysis. (**A**) Volcano plot depicting the direction, strength, and statistical significance of all 18,000 correlations examined in the analysis. Correlations were assed via Spearman’s rho and p-values were corrected via the Bonferroni method to account for multiple comparisons. (**B**) Relationship between total transcriptional expression levels of AKAP7 and total transcriptional expression levels of ITGA3 in the peripheral blood of AIS patients at emergency department admission. Expression levels are presented as mean normalized microarray florescence.

In order to gain insight into the possible role of AKAP7 in the context of post stroke BBB disruption, we next performed a genome-wide correlational analysis in which the correlation coefficients between the expression levels of *AKAP7* and each of the other 18,000 genes detected via microarray were calculated. Genes were then ranked based on the absolute strength of relationship between their expression levels and those of *AKAP7*. We theorized that genes which were strongly co-expressed with *AKAP7* would likely be mechanistically related. *ITGA3*, which encodes a cellular adhesion molecule known as integrin alpha 3 (ITGA3), was the only gene in the dataset whose expression levels were significantly correlated with those of *AKAP7* (Fig. 3A), exhibiting a robust positive association (Fig. 3B). Furthermore, like *AKAP7*, early expression levels of *ITAG3* at were positively associated with the development of post-stroke severe HARM in a post-hoc logistic regression analysis controlling for age, dyslipidemia, and rtPA (p = 0.045*, Odds ratio = 3.5, 95% confidence interval = 1.0–28.4). Due to the established role of ITGA3 in mediating leukocyte extravasation^19^, we hypothesized that AKAP7 may be functionally involved in ITGA3 mediated leukocyte trafficking post-stroke.

### Established and bioinformatically predicted AKAP7 splice variants are expressed in the peripheral immune system and appear to be inversely regulated

**Figure 4:**
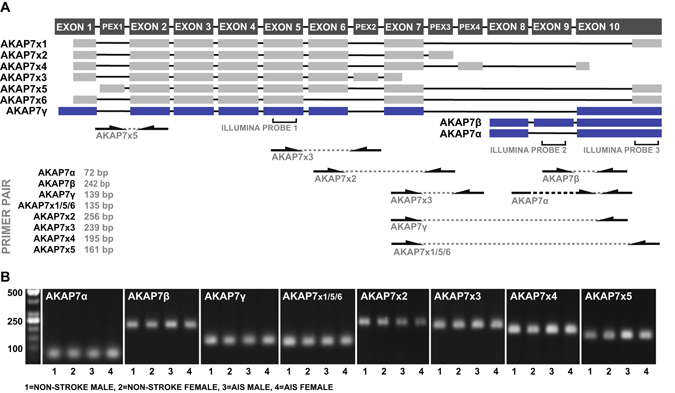
Detection of AKAP7 splice variants in peripheral blood. (**A**) Exon maps of previously validated (blue) and bioinformatically predicted (grey) AKAP7 splice variants with the location of microarray probes and variant-specific priming sites. PEX denotes a bioinformatically predicted exon. (**B**) AKAP7 splice variant-specific RT-PCR products amplified from peripheral blood-derived cDNA obtained from two non-stroke donors and two stroke patients.

It is well established that AKAP7 is subject to alternative splicing, however, little is known about the expression of these splice variants in the peripheral immune system. *AKAP7* codes for three splice variants which are known to generate functional scaffolding proteins (AKAP7α, AKAP7β, AKAP7γ) with varying biological activity within the context of PKA signaling^20^. In addition to these established splice variants, *AKAP7* may also code for as many as six bioinformatically predicated alternatively spliced transcripts (AKAP7x1, AKAP7x2, AKAP7x3, AKAP7x4, AKAP7x5, AKAP7x6)^21^. In order to determine which AKAP7 splice variants were responsible for the coregulatory relationship we observed with ITGA3, we first aimed to identify which AKAP7 splice variants are expressed in the peripheral immune system. Primers specific to each established and predicated splice variant were designed based on variant-specific priming sites (Fig. 4A) and RT-PCR was used to probe for the presence of expression in peripheral whole blood samples obtained from two stroke patients and two healthy subjects. PCR products were detected for all tested AKAP7 splice variants in all samples (Fig. 4B), providing direct evidence that all established and previously bioinformatically predicted AKAP7 splice variants are expressed in the peripheral immune system. Where possible, a second set of primers was used to validate the expression of previously predicted variants (Supplementary Figure 1A).

**Figure 5:**
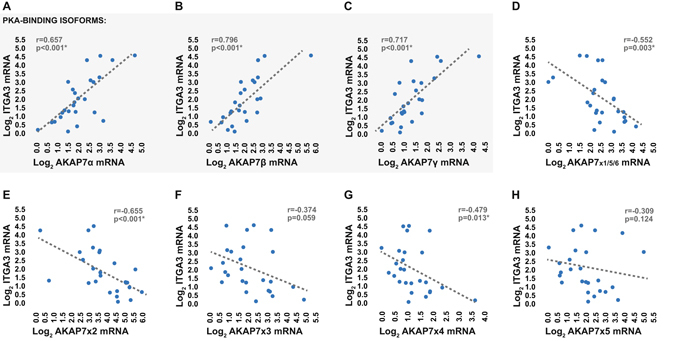
Relationship between peripheral blood expression levels of AKAP7 splice variants and ITGA3. (**A**–**H**) Relationship between transcriptional expression levels of AKAP7 splice variants and total transcriptional expression levels of ITGA3 in the peripheral whole blood of validation cohort AIS patients at emergency department admission as measured using qRT-PCR. Splice variants which are translated to produce PKA-binding AKAP7 isoforms are highlighted. Expression levels are presented as log_2_ fold differences relative to the sample of lowest expression.

Once we identified that all established and previously predicted AKAP7 splice variants are expressed in the peripheral immune system, the expression levels of each splice variant were measured in parallel with those of ITGA3 in the peripheral blood of an identically recruited independent validation cohort of AIS patients using qRT-PCR (Table 2). In this second cohort of patients, early expression levels of AKAP7α, AKAP7β, and AKAP7γ exhibited significant positive correlations with expression levels of ITGA3 (Fig. 5A–C). Interestingly, all previously predicted AKAP7 splice variants displayed inversely related expression levels with regards to ITGA3 (Fig. 5D–H) as well as the established AKAP7 splice variants (Supplementary Figure 2). These data suggest that the strong positive relationship between AKAP7 and ITGA3 expression observed in the first cohort of patients was primarily driven by AKAP7α, AKAP7β, and AKAP7γ. This is feasible due to the fact that the previously predicted AKAP7 splice variants appeared to be expressed at much lower levels than those of the established splice variants, as indicated by higher CT values in qRT-PCR (data not shown) and the production of significantly less PCR product in standard RT-PCR when using primers designed for co-amplification (Supplementary Figure 1B). Interestingly, AKAP7α, AKAP7β, and AKAP7γ all encode for scaffolding proteins with the ability to bind PKA^20^, while the remaining splice variants are predicted to undergo non-sense mediated decay or generate scaffolding proteins which lack a PKA binding domain (Supplementary Figure 3). Collectively, the data validate the relationship between ITGA3 and AKAP7 observed in the discovery cohort, and suggest dynamic regulation between the PKA-binding and non-PKA-binding AKAP7 variants. Thus, the ratio between the expression levels of these splice variants may be more diagnostically robust than the expression levels of total AKAP7 alone.

**Table 2:** Validation cohort clinical and demographic characteristics.

	n = 26
Age (*mean* ± *SD*)	70.3 ± 14.1
Female *n* (%)	16 (61.5)
Baseline NIHSS (*mean* ± *SD*)	8.0 ± 7.1
Partial anterior cerebral infarction *n* (%)	11 (42.3)
Total anterior cerebral infarction *n* (%)	4 (15.4)
Lacunar cerebral infarction *n* (%)	5 (19.2)
Posterior cerebral infarction *n* (%)	6 (23.1)
Hypertension *n* (%)	24 (92.3)
Diabetes *n* (%)	4 (15.4)
Atrial Fibrillation *n* (%)	10 (38.5)
Myocardial infarction *n* (%)	6 (23.1)
Dyslipidemia *n* (%)	12 (46.2)
Hypertension medication *n* (%)	22 (84.6)
Diabetes medication *n* (%)	4 (15.4)
Cholesterol medication *n* (%)	10 (38.5)
Anticoagulant/antiplatelet *n* (%)	8 (30.8)
rtPA *n* (%)	10 (38.5)
Current Smoker *n* (%)	6 (23.1)
Family History of stroke *n* (%)	11 (42.3)
Previous Stroke *n* (%)	6 (23.1)

SD, standard deviation; NIHSS, National Institutes of Health stroke scale; rtPA, recombinant tissue plasminogen activator.

### PKA-binding AKAP7 isoforms are highly expressed on lymphocytes in peripheral blood

**Figure 6:**
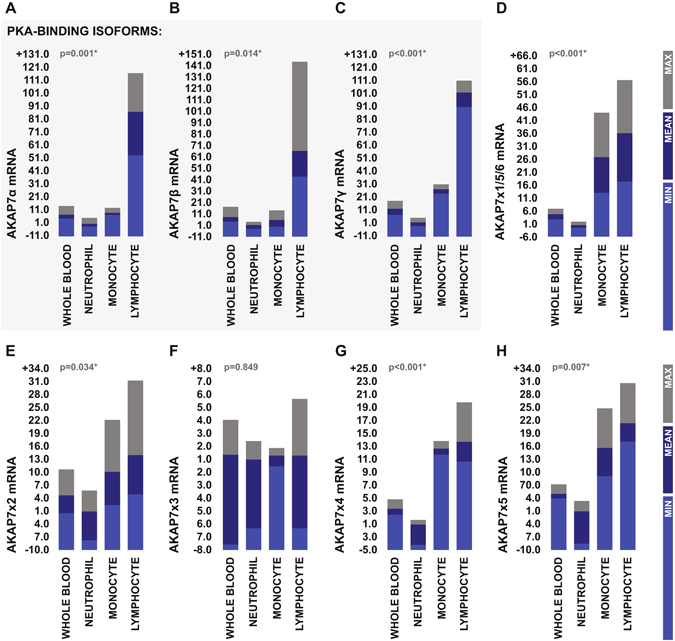
Expression of AKAP7 splice variants in primary leukocyte populations isolated from peripheral blood. (**A**–**H**) Transcriptional expression levels of AKAP7 splice variants in primary leukocyte populations isolated from the peripheral blood of two male and one female heathy donors as measured by qRT-PCR. Splice variants which are translated to produce PKA-binding AKAP7 isoforms are highlighted. Expression levels are presented as fold difference relative to the population of lowest expression and were statistically compared using one-way ANOVA.

To gain further insight into the possible functional role of AKAP7 in the context of post-stroke BBB disruption, we next looked to determine the cellular source of AKAP7 in the peripheral immune system. qRT-PCR was used to measure the expression levels of the AKAP7 splice variants in RNA extracted from isolated leukocyte subpopulations obtained from the peripheral blood of three healthy donors using immuno-magnetic negative selection. Expression levels of the PKA-binding AKAP7 isoforms appeared to be significantly higher on cells of lymphoid origin (Fig. 6), leading us to hypothesize that AKAP7 levels may serve as a marker of post-stroke lymphocyte trafficking specifically.

### AKAP7 expression is associated with a highly-adherent lymphocyte phenotype *in vitro*

**Figure 7:**
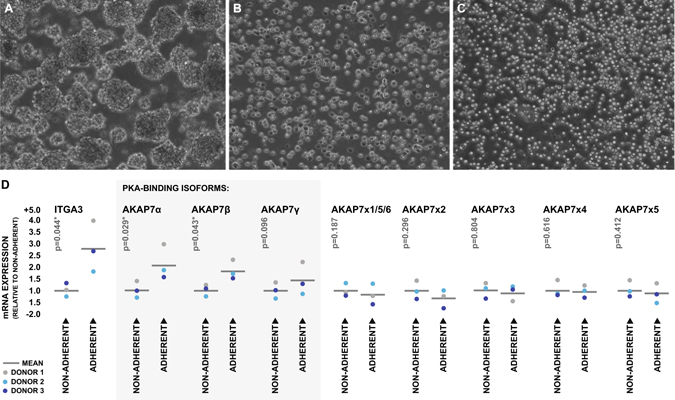
Expression of AKAP7 splice variants and ITGA3 on differentially adherent lymphocyte populations. (**A**) Actively proliferating primary human lymphocytes following 72 hours of *in vitro* stimulation with PHA. (**B**) Adherent fraction of lymphocytes bound to extracellular matrix rich in ITGA3 ligands following 20 minutes of serum starvation. (**C**) Non-adherent fraction of lymphocytes collected following 20 minutes of serum starvation. (**D**) Transcriptional expression levels of total ITGA3 and AKAP7 splice variants in adherent and non-adherent lymphocyte fractions as measured by qRT-PCR. Splice variants which are translated to produce PKA-binding AKAP7 isoforms are highlighted. Expression levels are presented as fold difference relative to the adherent fraction and were statistically compared using two-sample two-way paired t-test.

In order to test that hypothesis that AKAP7 may be a marker of lymphocyte extravasation, specifically by way of ITGA3-mediated adhesion, qRT-PCR was used to measure the transcriptional levels of AKAP7 splice variants and ITGA3 in primary human lymphocytes which exhibited a strong ability to adhere to culture surfaces coated in a matrix abundant in ITGA3 ligands. Lymphocytes were isolated from the peripheral blood of three healthy donors and expanded for 72 hours yielding healthy, actively proliferating lymphocytes (Fig. 7A). Lymphocytes were then plated in culture dishes coated in a matrix composed of collagen, laminin, and fibronectin under serum-free conditions and allowed 20 minutes for adherence. Non-adherent lymphocytes were collected from the culture supernatant (Fig. 7B), and the adherent lymphocytes were then separately harvested (Fig. 7C). Viability of cells in both fractions was assessed via trypan blue and RNA was extracted for gene expression analysis. Cell viabilities were greater than 95% in both populations in the case of every donor. Expression levels of ITGA3 and the PKA-binding AKAP7 variants were significantly higher on lymphocytes in adherent fractions than those in the non-adherent fractions (Fig. 7D). Collectively these data suggest that AKAP7 expression is a marker of ITGA3-mediated lymphocyte adhesiveness and is likely elevated in patients who later develop post-stroke BBB disruption as a result of the presence of an invasive lymphocyte population in peripheral blood.

## Discussion

The objective of this study was to determine whether the early expression levels of any of 16 candidate genes which we previously established as differentially regulated in AIS could be used to identify patients at high risk for post-stroke BBB disruption. Our results suggest that elevated expression levels of AKAP7 during the early acute phase of care may be predicative for the development of BBB-disruption in the days following injury. Furthermore, we show evidence that AKAP7 expression may be elevated in patients who later develop post-stroke BBB disruption as a result of the presence of an invasive lymphocyte population in peripheral circulation.

AKAP7 is a widely expressed scaffolding protein whose primary cellular function is spatial regulation of cyclic adenosine monophosphate (cAMP) signaling via cytoskeletal anchoring of PKA^22–24^. Alternative splicing results in multiple AKAP7 isoforms which are functionally diverse within the context of A-kinase signaling. The *AKAP7* gene codes for three splice variants (AKAP7α, AKAP7β, AKAP7γ) which are known to be expressed and produce functional protein isoforms (Supplementary Figure 3)^20^. These AKAP7 isoforms all contain a conserved PKA-binding domain, but distinctive tethering domains and targeting motifs which are associated with differing intracellular localization^20, 25^. AKAP7γ is unique with regards to AKAP7α and AKAP7β in that it contains a phosphodiesterase (PDE) domain^26^. The function of this domain is currently unclear; structural similarity to bacterial 2′5′ RNA ligases has led to the hypothesis that it may have RNA ligase activity^27^, while studies which have shown AMP binding ability have led to the suggestion that it serves as an AMP sensor^26^. In addition to the splice variants which are known to code for proteins, the National Center for Biotechnology Information (NCBI) eukaryotic genome pipeline had previously predicted that the *AKAP7* gene may also code for as many as six additional splice variants^21^. The sequences of these previously predicated splice variants suggest some would be targeted for non-sense mediated decay while others would generate protein isoforms containing the PDE domain but lacking the PKA binding domain (Supplementary Figure 3). While AKAP7 has been most widely studied in the skeletal muscle, cardiac tissue, and the brain, little is known about the physiological role of AKAP7 in the peripheral immune system.

In order to gain insight into the possible function of leukocyte AKAP7 in the context of post-stroke BBB disruption, we aimed to establish which AKAP7 splice variants are expressed in the peripheral immune system following stroke, determine what cell populations they are expressed on, as well as identify molecules which exhibit a pattern of co-regulation. Our results provided evidence that all previously established protein coding AKAP7 splice variants, as well as all previously predicted AKAP7 splice variants, are expressed in the peripheral immune system. Our results further suggest that the PKA-binding isoforms of AKAP7 are predominantly expressed on cells of lymphoid origin, and that their expression levels are highly correlated with those of ITGA3, a cell adhesion molecule known to interact with various extracellular matrix proteins including with laminin, collagen, and fibronectin^28^. ITGA3 has recently been shown to signal via cAMP and PKA upon ligand binding^29^; thus, it is highly plausible that the strong relationship we observed between the expression levels of the PKA-binding AKAP7 splice variants and the expression levels of ITGA3 exists because these AKAP7 isoforms mediate the interaction between ITGA3 and PKA. Interestingly, elevated expression of ITGA3 is associated with lymphocyte invasiveness^30, 31^, and inhibition of ITGA3 has been to shown to limit leukocyte extravasation in inflammatory conditions such as sepsis^19^. In light of our observations, this information led us to hypothesize that the PKA-binding isoforms of AKAP7 are markers of ITGA3-mediated lymphocyte adhesiveness, and are likely elevated in patients who later develop post-stroke BBB disruption as a result of the presence of an invasive lymphocyte population in the peripheral blood.

The results of our *in vitro* experiments supported this hypothesis, as the expression levels of the PKA-binding isoforms of AKAP7, along with the expression levels of ITGA3, were elevated on primary lymphocytes which exhibited a strongly adherent phenotype indicative of invasiveness. The presence of a lymphocyte population with such properties in the peripheral immune system following stroke could contribute to disruption of the BBB by both direct and indirect mechanisms. The process of cerebrovascular lymphocyte extravasation alone inherently reduces the integrity of the BBB^32^, and heightened accumulation of lymphocytes in the brain parenchyma increases the risk of further BBB disruption via the development an adaptive immune response directed at central nervous system antigens^33^. The development of such a response leads to lymphocyte-mediated production of pro-inflammatory chemokines which drive increased influx of innate immune cell populations such as neutrophils and monocytes from the periphery into the central nervious system^34^; during this process, these myeloid immune populations produce proteolytic molecules such as matrix metalloproteinases which further act to compromise the integrity of the BBB^2^. Expectedly, it has been shown in multiple studies that increased lymphocyte activity post-stoke leads to poor prognosis^35, 36^. Thus, our results suggest that AKAP7 and ITGA3 are molecules which could potentially be targeted therapeutically to limit lymphocyte-mediated post-stroke injury exacerbation.

Additionally, our findings suggest that AKAP7 may be a clinically useful biomarker. Early identification of patients at heightened risk for post-stroke BBB disruption allows for clinicians to make more informed decisions regarding the administration of thrombolytic therapies, and can ultimately improve clinical management^6^. As a result, several prior studies have looked to identify blood biomarkers which are predicative for the development of post-stroke BBB disruption. The most widely investigated and once considered the most promising of these potential biomarkers is S100 calcium binding protein B (S100B). Multiple studies have observed early elevated plasma levels of S100B in AIS patients who later develop BBB disruption^37, 38^, however S100B has only exhibited modest levels of diagnostic robustness; in the largest clinical study performed to date evaluating the ability of S100B to identify patients at risk for post-stroke BBB disruption, S100B was only able to do so with 93% sensitivity and 48% specificity^38^. In the study reported here, albeit in a limited sample size, early transcriptional levels of AKAP7 exhibited an ability to predict post-stroke BBB disruption with levels of diagnostic performance exceeding those reported with regards to a majority of previously proposed biomarkers; these findings make AKAP7 a candidate for further clinical evaluation with regards to biomarker use.

In addition to providing insight into the role of leukocyte AKAP7 in the context of stroke, this study generated novel data regarding the genomic regulation of AKAP7. This study was the first to provide substantial evidence that all six AKAP7 splice variants which were bioinformatically predicted by the NCBI eukaryotic genome pipeline are expressed in nature. Of these variants, AKAP7x3 and AKAP7x6 are predicted to undergo non-sense mediated decay based on the presence of stop codons more than 50 nucleotides upstream of the first 3′ exon-exon junction^39^. The sequences of remaining previously predicated splice variants (AKAP7x1, AKAP7x2, AKAP7x4, AKAP7x5) suggest they have the capacity to code for proteins. The protein isoforms which would be generated by these splice variants would be highly homologous to AKAP7γ, however lack the presence of the PKA binding domain. This suggests it is likely that AKAP7 protein isoforms exist in nature which lack A-kinase binding ability. It is possible that these isoforms could act as decoy proteins which occupy AKAP7 anchoring sites to inhibit the scaffolding of A-kinases. Alternatively, because three of these four potential protein isoforms are predicted to contain an untruncated PDE domain as their predominant feature, it is also plausible that they have some other biological function directly inferred by the PDE domain. As future studies provide more insight regarding the role of the PDE domain, the function of these previously predicated splice variants may become more clear. Our results imply that these previously predicted splice variants are expressed at much lower levels in the peripheral immune system than the established splice variants which code for PKA-binding protein isoforms, however, the dynamic intra-variant regulation we observed suggests they are of biological significance. Expectedly, due to their seemingly opposing biological function within the context of A-kinase signaling, we observed inversely regulated expression levels between the previously predicted splices variants and the established splice variants which code for PKA-binding protein isoforms. Thus, AKAP7 alternative splicing may play a much larger role in regulation of cAMP signaling than previously anticipated.

Due to the preliminary nature of this study, it is important to note that it is not without limitations. The sample size in this study was not large enough to draw definitive conclusions regarding the potential use of AKAP7 as a clinical biomarker for prediction of post-stroke BBB complications. However, we feel the results of this this preliminary analysis are promising and investigation into the prognostic value of AKAP7 in a larger clinical cohort is warranted. In terms of understanding the mechanistic role of AKAP7 in the peripheral immune system with regards to stroke pathophysiology, much of the analysis in this study was associative in nature and performed only at the level of transcription. The biological mechanisms proposed based on the associative observations of this study should be validated in future pre-clinical work which takes a more direct experimental approach, potentially via genetic manipulation of AKAP7 *in-vivo*. Furthermore, our assumption that the association between whole blood AKAP7 levels and post-stroke BBB disruption is driven by a lymphocyte-dependent mechanism was based on the observation of lymphocyte-specific AKAP7 expression on isolated leukocyte subpopulations; however, this analysis was restricted to a limited number of healthy individuals and only surveyed the three most predominant subpopulations of leukocytes. Thus, future work may want to address the possibility that non-lymphoid sources of AKAP7 may contribute to the whole blood elevation in expression levels observed with post-stroke BBB disruption. Despite the aforementioned limitations, we feel that this work provides compelling evidence that AKAP7 in the peripheral immune system plays a role in post-stroke BBB pathology. We feel these results provide valuable foundational knowledge which will allow for more in-depth exploration into AKAP7 as both a clinical biomarker and therapeutic target with regards to post-stroke BBB disruption.

Collectively, our results suggest that early expression levels of AKAP7 in the peripheral immune system are predictive of the development of BBB disruption in the days following ischemic stroke. Furthermore, our findings suggest that AKAP7 is a marker for a highly adherent lymphocyte phenotype, and may be elevated in patients who later develop post-stroke BBB disruption as a result of the presence of an invasive lymphocyte phenotype in peripheral blood. Thus, AKAP7 may a clinically useful biomarker for the identification of patients at heightened risk for the development of post-stroke BBB disruption. This work yields novel insight regarding the biology of AKAP7 in the context of the peripheral immune system, and provides a foundation for further study regarding the role of AKAP7 in stroke pathology.

## Methods

### Discovery Cohort Patients

Discovery cohort AIS patients were recruited at Suburban Hospital (Bethesda, MD). All AIS patients displayed definitive radiographic evidence of vascular ischemic pathology on MRI according to the established criteria for diagnosis of acute ischemic cerebrovascular syndrome (AICS)^40^, and diagnoses were confirmed by an experienced neurologist. Prospective subjects were excluded if they displayed radiographic evidence of BBB disruption at emergency department admission, reported a prior hospitalization within 90 days, were under 18 years of age, or were admitted more than 24 hours post-symptom onset. Type of cerebral infarction was classified via the four categories described by Bamford *et al*.^41^ based solely on radiographic evidence of the following^42^: anterior circulation infarct with restricted cortical involvement (partial anterior cerebral infarction), large anterior circulation infarct with both cortical and subcortical involvement (total anterior cerebral infarction), small perforating artery infarction (lacunar cerebral infarction), vertebrobasilar territory infarction (posterior cerebral infarction). Time from symptom onset was determined by the time the patient was last known to be free of AIS symptoms. Injury severity was determined according to the National Institutes of Health stroke scale (NIHSS) at the time of blood draw. Demographic information was collected from either the subject or significant other by a trained clinician. All procedures were approved by the institutional review boards of the National Institute of Neurological Disorders/National Institute on Aging at the National institutes of Health and Suburban Hospital. All experiments were performed in accordance with relevant guidelines and regulations. Informed consent was obtained from all subjects or their authorized representatives prior to any study procedures.

### Validation cohort patients

Validation cohort AIS patients were recruited at Ruby Memorial Hospital (Morgantown, West Virginia). All AIS patients displayed definitive radiographic evidence of vascular ischemic pathology on either computed tomography (CT) or MRI according to the same AICS diagnostic criteria^40^ utilized for discovery cohort recruitment. Patient exclusion, stroke sub-type classification, assessment of injury severity, and collection of demographic information was performed in an identical manner as it was with regards to the discovery cohort. All procedures were approved by the institutional review boards of West Virginia University and Ruby Memorial Hospital. All experiments were performed in accordance with relevant guidelines and regulations and informed consent was obtained from all subjects or their authorized representatives prior to any study procedures.

### Magnetic resonance imaging for assessment of HARM

MRI was performed on discovery cohort patients using a 1.5-Tesla clinical magnetic resonance system both at emergency department admission and at 24 hour follow-up. The standardized protocol included: diffusion-weighted imaging, T2-weighted gradient-recalled echo, FLAIR, and perfusion-weighted imaging. Perfusion-weighted imaging was obtained using a bolus passage of Gd-DTPA (0.1 mmol/kg). FLAIR images were assessed for location and level of HARM as previously described^8^. HARM was positivity identified by the post-contrast appearance of CSF hyperintensity in the sulci or ventricles (Fig. 1). Level of HARM was classified based on the following criteria: no hyperintensity (none), punctate regions of hyperintensity (mild), continuous regions of hyperintensity on 1–10 consecutive images (moderate), continuous regions of hyperintensity on >10 consecutive images (severe). Images were reviewed sequentially by expert readers, blinded to clinical information.

### Blood collection and RNA extraction

Peripheral blood samples were collected via PAXgene RNA tubes (Qiagen, Valencia, CA) and stored at −80 °C until RNA extraction. RNA was extracted via PreAnalytiX PAXgene blood RNA kit (Qiagen) and automated using the QIAcube system (Qiagen). Quantity and purity of isolated RNA was determined via spectrophotometry (NanoDrop, Thermo Scientific, Waltham, MA). Quality of RNA was confirmed by chip capillary electrophoresis (Agilent 2100 Bioanalyzer, Agilent Technologies, Santa Clara, CA).

### Microarray

RNA was amplified and biotinylated using the TotalPrep RNA amplification kit (Applied Biosystems, Grand Island, NY). Samples were hybridized to HumanRef-8 expression bead chips (Illumina, San Diego, CA) and scanned using the Illumina BeadStation. Raw probe intensities were background subtracted, quantile normalized, and then summarized at the gene level (total gene expression) using Illumina GenomeStudio. Sample labeling, hybridization, and scanning were performed per standard Illumina protocols. mRNA expression values are presented as normalized mean probe intensity. Raw data are available via NCBI Gene Expression Omnibus (GEO) accession GSE16561.

For genome-wide correlational analysis, correlation coefficients between the expression levels of genes identified as being predictive of severe HARM and each of the remaining genes detected via microarray were calculated. Genes were then ranked based on the absolute strength of relationship between their expression levels and those of HARM-associated genes. Statistical significance of relationships was evaluated via two-tailed critical testing with Bonferroni correction to account for multiple comparisons.

### RT-PCR

cDNA was synthesized using the Applied Biosystems High Capacity Reverse Transcription Kit. For quantitative PCR, target sequences were amplified from 10 ng of cDNA input using custom probe sets designed using the NCBI primer design tool and detected via SYBR green (PowerSYBR, Thermo-Fisher) on the RotorGeneQ (Qiagen). Raw amplification plots were background corrected and CT values were generated via the RotorGeneQ software package. All reactions were performed in triplicate. Transcripts of *B2M*, *PPIB*, and *ACTB* were amplified as low variability references and normalization was performed using the NORMAgene data-driven normalization algorithm^43^. Expression values are presented as fold difference relative to control in the case of groupwise comparisions, or as fold value relative to lowest expression for correlational analysis.

For non-quantitative PCR applications, 100 ng of template was amplified using ReddyMix Complete master mix (Thermo Scientific) and products were visualized via ethidium bromide following agarose gel electrophoresis. Primer sequences and thermocycling conditions are listed in Supplementary Table 1.

### Isolation of Leukocyte Sub-populations

Blood was collected from healthy donors via K_2_ EDTA vacutainer and leukocytes were isolated via immuno-magnetic negative selection (EasySep Direct, StemCell Technologies, Vancouver, CA). Neutrophils, total lymphocytes, and monocytes were isolated from 4 mLs each of EDTA-treated blood per manufacture instructions. Isolated cells were rinsed once in PBS and lysed in Qiagen buffer RLT containing beta-mercaptoethanol, flash frozen in liquid nitrogen, and stored at −80 until RNA extraction.

### Primary lymphocyte culture and adhesion assay

All cell culture was performed using aseptic technique under standard mammalian culture conditions. For isolation of primary lymphocytes, 10 mLs of whole peripheral blood was collected via EDTA vacutainer and peripheral blood mononuclear cells were isolated via density gradient centrifugation. Two rounds of differential plating were performed to separate monocytes from lymphocytes. Lymphocytes were expanded for 72 hours in a growth media comprised of RPMI-1640 (Life Technologies, Grand Island, NY) containing 10% autologous human serum, beta-mercaptoethanol (Gibco, Grand Island, NY), phytohaemagglutinin (PHA, Gibco), and HEPES (Gibco). For adhesion assays, expanded lymphocytes were suspended in serum-free media and plated in 6-well culture vessels coated in a matrix comprised of recombinant human collagen, fibronectin, and laminin (Gibco). After 20 minutes, non-adherent cells were collected from the culture supernatant via centrifugation and the remaining adherent cells were separately harvested via scraping. Cell fractions were lysed in Qiagen buffer RLT and stored at −80 for later RNA extraction. Adherent and non-adherent fractions from identical cultures were used for viability testing via trypan blue. Data represent lymphocyte populations obtained from three healthy donors, all assayed in triplicate.

### Statistics

Statistical analysis was performed using SPSS (IBM, Chicago, Ill) in combination with R 2.14 (R project for statistical computing) via the SPSS R integration plug-in. Chi-squared test was used for comparison of dichotomous variables. Student t-test or one-way ANOVA was used for comparison of continuous variables where appropriate. Spearman’s rho was used to assess the strength of correlational relationships. Bias-reduced logistic regression was performed using the logistf R package^44^; regression was performed using standardized gene expression levels, and model inclusion of independent variables was determined via forward stepwise selection. Receiver operator characteristic (ROC) analysis was used to test the performance of binary classifiers; optimal cutoff value was determined by the cutoff which yielded the greatest level of combined sensitivity and specificity. The level of significance was established at 0.05 for all statistical testing. P-values were corrected via the Bonferroni method in the case of multiple comparisons.

## Electronic supplementary material

Supplemantary Materials
