# Supplementary material for: Peripheral blood AKAP7 expression as an early marker for lymphocyte-mediated post-stroke blood brain barrier disruption
Source: Sci Rep. 2017 Apr 26;7:1172. doi: 10.1038/s41598-017-01178-5 (PMC5430856; doi:10.1038/s41598-017-01178-5)
Supplement: Supplementary file 1 — Supplemantary Materials [file 41598_2017_1178_MOESM1_ESM.pdf]

SUPPLEMENTARY MATERIAL

Peripheral blood AKAP7 expression as a marker for lymphocyte mediated post-stroke blood brain barrier disruption

Grant C. O'Connell, PhD; Madison B. Treadway, BSc; Ashley B. Petrone, PhD; Connie S. Tennant, RN; Noelle Lucke-Wold, RN; Paul D. Chantler, PhD; Taura L. Barr, RN, PhD

Supplementary Figures: 3
Supplementary Tables: 1

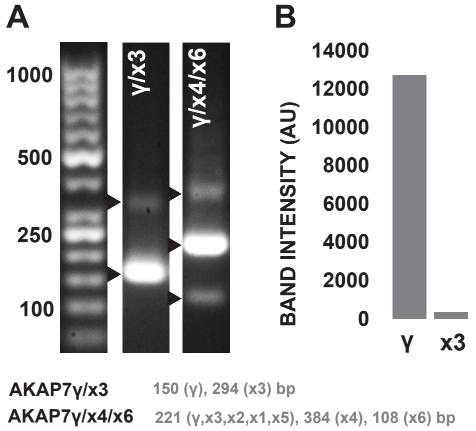

SUPPLEMENTARY FIGURE 1. *Validation of predicted splice variant expression in peripheral blood.*

(A) Alternate primer pairs designed to co-amplify multiple splice variants were used to confirm the expression of AKAP7x3, AKAP7x4, and AKAP7x6 in peripheral blood. Arrowheads indicate expected products. (B) Comparison of the band intensity of PCR products generated by coamplification of AKAP7y and AKAP7x3 as quantified by ImageJ. Significantly more AKAP7y PCR product was detected following co-amplification.

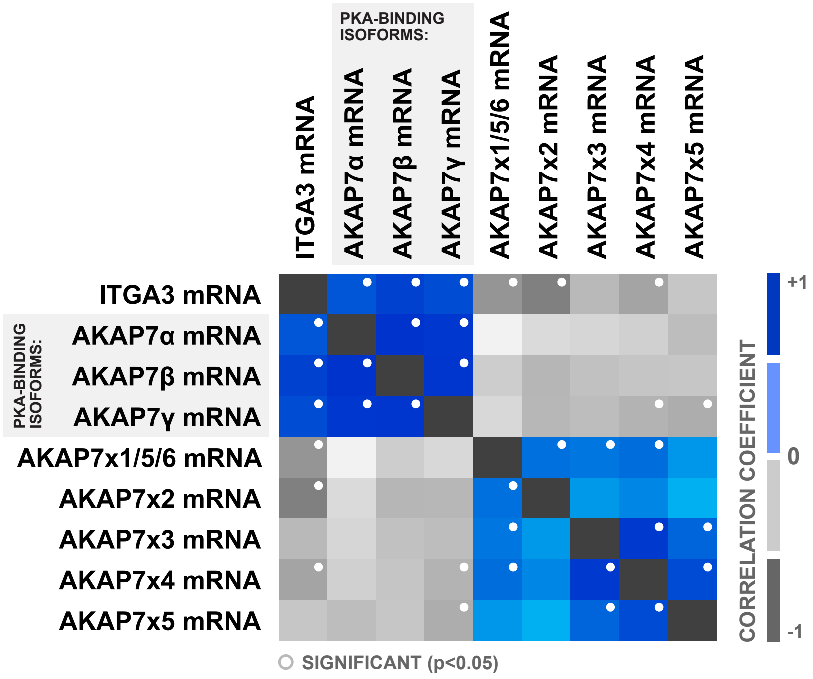

SUPPLEMENTARY FIGURE 2. *Relationship between the expression levels of AKAP7 splice variants in peripheral blood following AIS.*

Correlation matrix depicting the relationships between the expression levels of individual AKAP7 splice variants following AIS. Splice variants which are known to code for PKA-binding AKAP7 isoforms are highlighted.

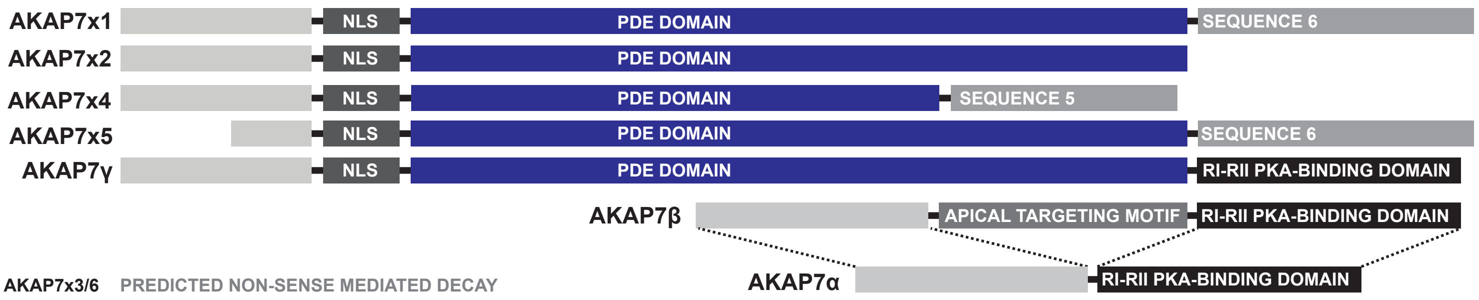

SEQUENCE 5:
PRGFLMQIQT SDGLLSYTRV LEMGMQINIR SLLSMTLCCS

SEQUENCE 6:
KKPIGIRDLI NEALHRETMG LKSKVKQIKE LLLKPETQAR IRRELFEGRL INNSNSANDV DFSTTLT

SUPPLEMENTARY FIGURE 3. *Alignment of established AKAP7 protein isoforms and possible protein isoforms encoded by previously predicted AKAP7 splice variants.*

Protein sequences were generated from RefSeq RNA sequences using the ExPASy translate tool. AKAP7x3 and AKAPx6 are predicted to undergo non-sense mediated decay based on the presence of stop codons more than 50 nucleotides upstream of the first 3’ exon-exon junction. If translated, the remaining previously predicted splice variants would generate protein isoforms highly homologous to AKAP7γ, but lack the presence of the PKA-binding domain. Some of these hypothetical isoforms would contain amino acid sequences which are not found on any of the three established protein isoforms. A conserved domain search failed to identify any conserved biological function for these sequences.

SUPPLEMENTARY TABLE 1. *Primers and thermocycling conditions used for RT-PCR.*

TARGETS	TRANSCRIPTS[1]	PRIMERS (5' to 3')[2]		PRODUCTS (bp)
**AKAP7α**	NM_004842.3	**FOR:**	GGAAAAATCAGTGAAAAGAACGGA	**72**
		**REV:**	CACCAGCCTCTTACTGAGCC	
**AKAP7β**	NM_138633.2	**FOR:**	GCAGCAAGTTCCCTTCTCCTT	**242**
		**REV:**	TTTCCAACTCACTGATTTTTCCTTC	
**AKAP7γ**	NM_016377.3	**FOR:**	ATTGTGATTGGTGAAAAGAACGGAG	**139**
		**REV:**	CCCCCGGCTTGTTTTTATTCT	
**AKAP7x1**	XM_005267228.1	**FOR:**	TAGATCTTTGCTCCATGCTGAA	**135**
**AKAP7x5**	XM_005267229.3	**REV:**	GACCCATCGTTTCTCGATGC	**135**
**AKAP7x6**	XR_427979.1			**135**
**AKAP7x2**	XM_011536262.1	**FOR:**	ACCGTGGCTCCGTAAGAATG	**256**
		**REV:**	CACTGTTCCCTCTTCTAAAATGCT	
**AKAP7x3**	XR_942636.1	**FOR:**	CCGTGGCTCCGTAAGAATGG	**239**
		**REV:**	CAGTTGCTCACACTCAGTCC	
**AKAP7x4**	XM_006715614.2	**FOR:**	GGTTGGATTTGTGAAGCTGGC	**195**
		**REV:**	ATTTGCATGAGGAACCCCCTA	
**AKAP7x5**	XM_005267229.3	**FOR:**	AAAGGAAAGCCTGGAGGTCAA	**161**
		**REV:**	GGCATGTCTACCAGAGAATCCAT	
**AKAP7γ**	NM_016377.3	**FOR:**	GAAGCTGGCAGAAGGAGATCA	**221**
**AKAP7x1**	XM_005267228.1	**REV:**	CTTCTCCAAATCTGTGACTGATAAA	**221**
**AKAP7x2**	XM_011536262.1			**221**
**AKAP7x3**	XR_942636.1			**221**
**AKAP7x4**	XM_006715614.2			**384**
**AKAP7x5**	XM_005267229.3			**221**
**AKAP7x6**	XR_427979.1			**108**
**AKAP7γ**	NM_016377.3	**FOR:**	ATCAGTCACAGATTTGGAGAAGA	**150**
**AKAP7x3**	XR_942636.1	**REV:**	TACTAGTTCAGCGTCATCGGG	**194**
**TOTAL ITGA3**	NM_002204.3	**FOR:**	CTACCACAACGAGATGTGCAA	**102**
	XM_005257308.1	**REV:**	CCGAAGTACACAGTGTTCTGG	**102**
**TOTAL PPIB**	NM_000942.4	**FOR:**	AAGTCACCGTCAAGGTGTATTTT	**153**
		**REV:**	TGCTGTTTTTGTAGCCAAATCCT	
**TOTAL B2M**	NM_004048.2	**FOR:**	GAGGCTATCCAGCGTACTCCA	**248**
	XM_006725182.2	**REV:**	CGGCAGGCATACTCATCTTTT	**248**
	XM_005254549.2			**248**
**TOTAL ACTB**	NM_001101.3	**FOR:**	CATGTACGTTGCTATCCAGGC	**250**
	XM_006715764.1	**REV:**	CTCCTTAATGTCACGCACGAT	**250**

[1]Listed by NCBI accession number

[2]All targets were amplified for 40 cycles of 95ºC (15s) / 60ºC (60s)
